# Multiple Regulatory Levels of Growth Arrest-Specific 6 in Mucosal Immunity Against an Oral Pathogen

**DOI:** 10.3389/fimmu.2018.01374

**Published:** 2018-06-18

**Authors:** Maria Nassar, Yaara Tabib, Tal Capucha, Gabriel Mizraji, Tsipora Nir, Faris Saba, Rana Salameh, Luba Eli-Berchoer, Asaf Wilensky, Tal Burstyn-Cohen, Avi-Hai Hovav

**Affiliations:** ^1^Faculty of Dental Medicine, The Institute of Dental Sciences, Hebrew University, Jerusalem, Israel; ^2^Department of Periodontology, Faculty of Dental Medicine, Hadassah Medical Center, Jerusalem, Israel

**Keywords:** growth arrest-specific 6, infection, oral, mucosa, immunoregulation

## Abstract

Growth arrest-specific 6 (GAS6) expressed by oral epithelial cells and dendritic cells (DCs) was shown to play a critical role in the maintenance of oral mucosal homeostasis. In this study, we demonstrate that the induction of pathogen-specific oral adaptive immune responses is abrogated in *Gas6*^−/−^ mice. Further analysis revealed that GAS6 induces simultaneously both pro- and anti-inflammatory regulatory pathways upon infection. On one hand, GAS6 upregulates expression of adhesion molecules on blood vessels, facilitating extravasation of innate inflammatory cells to the oral mucosa. GAS6 also elevates expression of CCL19 and CCL21 chemokines and enhances migration of oral DCs to the lymph nodes. On the other hand, expression of pro-inflammatory molecules in the oral mucosa are downregulated by GAS6. Moreover, GAS6 inhibits DC maturation and reduces antigen presentation to T cells by DCs. These data suggest that GAS6 facilitates bi-directional trans-endothelial migration of inflammatory cells and DCs, whereas inhibiting mucosal activation and T-cell stimulation. Thus, the orchestrated complex activity of GAS6 enables the development of a rapid and yet restrained mucosal immunity to oral pathogens.

## Introduction

The oral cavity is a unique anatomical structure composed of soft and hard tissues, which are continuously exposed to the microbiota. To ensure oral health, the immune system must maintain a symbiotic relationship with the microbiota while preventing invasion of pathogens. This task, however, is not trivial and oral pathogens such as *Porphyromonas gingivalis* can cause a disease by inducing microbial dysbiosis (1). Such an alteration in the composition of oral microbiota results in the development of destructive local immunity, which is associated with systemic adverse complications (2). This demonstrates the delicate equilibrium between the host immune system and microbiota, which likely requires sophisticated mechanisms to maintain a balanced homeostasis.

We recently reported the critical role of growth arrest-specific 6 (GAS6) protein in regulating oral mucosal homeostasis (3). GAS6 and Protein S (PROS1) are ligands of the TYRO3, AXL, and MERTK (TAM) receptor tyrosine kinases (4), which were found to play a role in various biological process including immunoregulation (5). In the oral mucosa, GAS6 and its predominant receptor AXL are expressed by the outermost layers of the epithelium. The expression of GAS6 is induced postnatally by the developing microbiota in a MYD88-dependent fashion (3). GAS6, in turn, downregulates the activation of epithelial cells, which is required for the establishment of oral mucosal homeostasis. Moreover, GAS6 expressed by dendritic cells (DCs) was found to restrain IL-6 production favoring the generation of T regulatory (Treg) over Th17 cells, further facilitating tolerogenic immune responses against the oral microbiota (3). Whereas these findings highlight a central role of GAS6/AXL signaling in regulating oral mucosal homeostasis, targeting the GAS6/AXL axis by pathogens might induce dysbiosis and subsequent oral pathology. Indeed, *P. gingivalis* is capable of degrading MYD88 in the oral epithelium resulting in a diminished expression of GAS6, AXL, and PROS1 (6). This leads to an oral microbial dysbiosis, elevated production of IFN-α by epithelial cells and temporal “immune paralysis” of the gingiva due to a lack of GAS6 and AXL expression in gingival blood vessels. Furthermore, the unrestrained IFN-α secretion due to the absence of negative regulation by GAS6 and AXL enables the development of excessive Th1-type inflammatory responses which enhance alveolar bone loss (6).

GAS6 thus emerges as a fundamental regulator of oral mucosal immunity at steady state, but also as a potential target for immune dysregulation by oral pathogens. Understanding the precise role of GAS6 during infection is therefore important in order to prevent or modulate local immunity for better protection. Nevertheless, GAS6 is widely expressed by many types of cells in the oral mucosa such as epithelial cells, DCs, and endothelial cells, making it difficult to precisely elucidate its function. This study is aimed at dissecting the mechanisms by which GAS6 engages distinct immunological phases in the oral mucosa upon infection with an oral pathogen.

## Results

### Diminished Pathogen-Specific Adaptive Immune Responses in *Gas6*^−/−^ Mice

**Figure 1:**
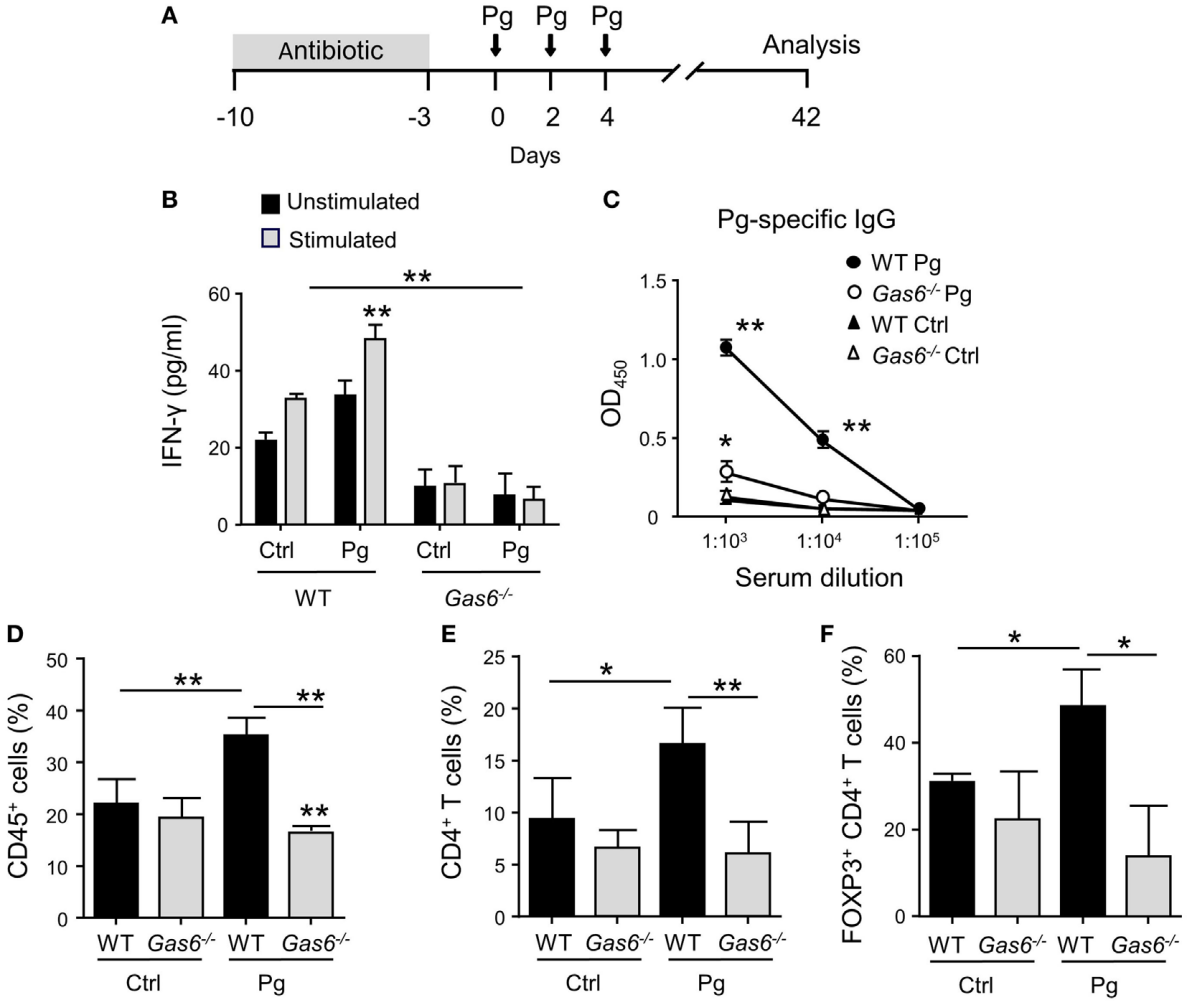
Diminished pathogen-specific adaptive immunity in *Gas6*^−/−^ mice. **(A)** Schematic presentation of the murine periodontitis model used in this study. Antibiotic pre-treated mice were infected *via* oral gavage, three times at 2-day intervals, with 1 × 10^10^ CFU of *Porphyromonas gingivalis* strain 53977 (Pg). Six weeks after the last inoculation, the mice were analyzed. **(B)** IFN-γ production by restimulated splenocytes quantified by ELISA representing the mean value ± SEM (*n* = 8). **(C)** Pg-specific IgG titers measured by ELISA in the plasma of the mice, representing the mean of OD_450_ values ± SEM (*n* = 8). **(D–F)** Flow cytometry analysis of the percentages of CD45^+^ cells **(D)**, CD4^+^ T cells **(E)**, and FOXP3-expressing CD4^+^ T regulatory cells **(F)** in the gingival tissues of infected mice (*n* = 5). Representative data of one out of three independent experiments is shown.

To study the role of GAS6 under inflammatory conditions, we employed the widely used oral infection model causing murine periodontitis. In this model, mice pre-treated with antibiotics are infected three times *via* oral gavage, with the oral pathogen *P. gingivalis* in a carboxymethylcellulose (CMC) solution as a vehicle (Figure 1A). To this end, *Gas6*^−/−^ mice and littermate *Gas6^+/+^* control (WT) mice were infected with *P. gingivalis* or vehicle and analyzed 6 weeks after the last infection. First, we assessed the development of Th1-type immune response by quantifying IFN-γ levels in the supernatants of splenocytes purified from the infected mice and restimulated *ex vivo* with the *P. gingivalis* antigen RgpA. As demonstrated in Figure 1B, significant secretion of IFN-γ was detected only in splenocytes of *P. gingivalis*-infected WT mice. Interestingly, besides the inability of restimulated splenocytes of *Gas6*^−/−^ mice to secrete IFN-γ, their baseline secretion of this cytokine was considerably lower in comparison to WT mice. Analyzing *P. gingivalis*-specific antibodies in the serum by ELISA revealed a similar phenomenon. *P. gingivalis*-infected WT mice contained a high circulating antibody titer whereas limited antibody titers were detected in infected and naïve *Gas6*^−/−^ mice (Figure 1C). We next examined the induction of oral mucosal immune responses by analyzing the gingiva of the infected mice. Using flow cytometry, we found higher frequencies of CD45^+^ leukocytes in the oral mucosa of *P. gingivalis*-infected WT mice compared to vehicle-treated mice (Figure 1D). In *Gas6*^−/−^ mice, on the other hand, no alteration in the percentages of these cells were detected. Further analysis identified an increase in CD4^+^ T cells in the gingiva of infected WT mice, while a fraction of these cells were Treg cells based on their ability to express the transcription factor FOXP3 (Figures 1E,F).

**Figure 2:**
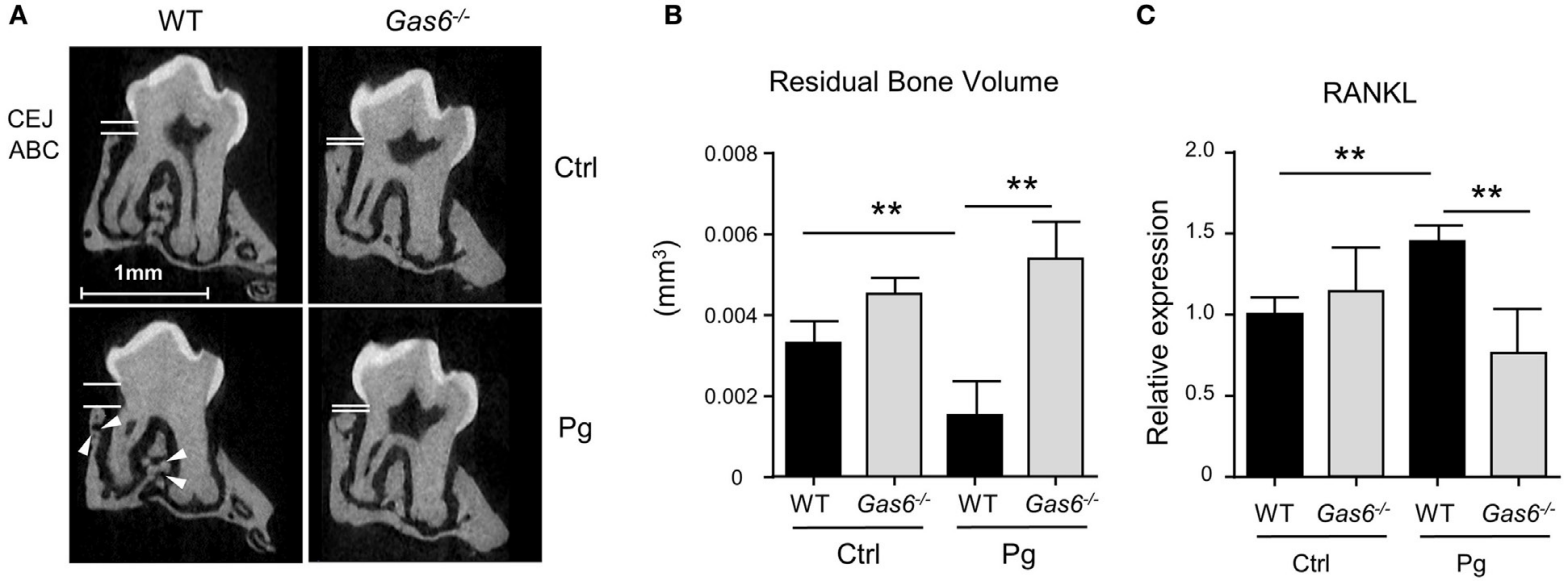
Lack of inflammation-induced bone loss in *Gas6*^−/−^ mice. **(A)** Representative μCT sections of the second upper molar demonstrating the residual bone volume measured from the cemento-enamel junction (CEJ) and alveolar bone crest (ABC). White arrows indicate lesions in the alveolar bone. **(B)** Three-dimensional quantification of the residual alveolar bone. Data are presented as the volume of alveolar bone in the buccal plate and represent the mean of eight mice per group ± SEM. **(C)** Quantification by RT-*q*PCR of the expression levels of RNAKL in the gingiva of infected mice, graphs represent the mean of five mice per group ± SEM. Representative data of one out of two independent experiments is shown.

The inflammatory responses induced in the gingiva following *P. gingivalis* infection are known to cause alveolar bone loss (7). We therefore quantified residual alveolar bone volume using μCT. Concurring with the above immunological data, a significant loss in the alveolar bone was observed only in *P. gingivalis*-infected WT mice, whereas no loss was found in *Gas6*^−/−^ mice (Figures 2A,B). Of note, care should be taken with the interpretation of these results, as GAS6 was reported to play a role in osteoclast activation *in vitro* (8). Nevertheless, the bone loss results were further supported using RT-*q*PCR analysis of RANKL expression (receptor activator of nuclear factor κ-B ligand). This molecule is known to be involved in *P. gingivalis*-induced alveolar bone loss and its expression significantly increased in WT but not *Gas6*^−/−^ mice due to the infection (Figure 2C). Taken together, these data demonstrate that GAS6 is essential to elicit *P. gingivalis*-specific adaptive immunity after oral infection.

### Differential Regulation of Inflammatory Leukocytes Versus APCs in Infected *Gas6*^−/−^ Mice

**Figure 3:**
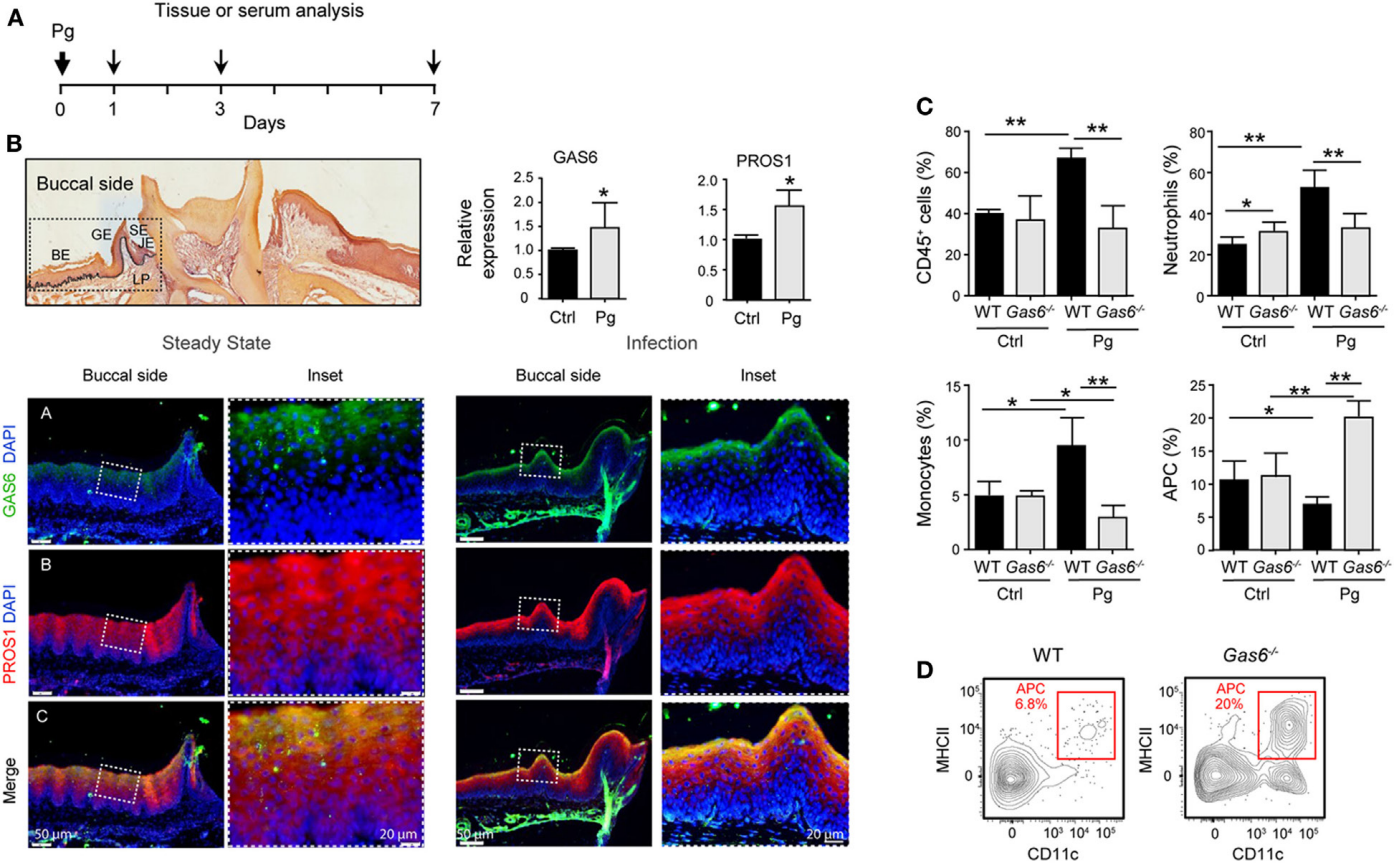
Lack of innate infiltrate in the gingiva of infected *Gas6*^−/−^ mice. **(A)** Schematic presentation of an acute oral infection. Mice were infected once with Pg by oral gavage and analyzed either 1, 3, or 7 days after infection. **(B)** H&E and immunofluorescence stained histological section of the lower jaw and RT-*q*PCR analyses of GAS6 and PROS1 expression in the gingiva of WT mice prior to and 3 days after Pg infection. Bar graphs represent the mean of four mice per group ± SEM. Immunohistochemical staining is shown for GAS6 (green), PROS1 (red) and nuclei are stained with hoechst (blue). Enlarged images of the framed area are shown to the right. **(C)** Percentages of total CD45^+^ cells, neutrophils, monocytes, and APCs in the gingiva 24 h after infection. Bar graphs represent the mean of five mice per group ± SEM. **(D)** Representative FACS plot showing the frequencies of MHCII^+^CD11c^+^ cells (APCs) in the gingiva of WT and *Gas6*^−/−^ mice 24 h after infection. Representative data of one out of three independent experiments is shown. Abbreviations: BE, buccal epithelium; GE, gingival epithelium; SE, sulcular epithelium; JE, junctional epithelium; LP, lamina propria; GAS6, growth arrest-specific 6.

To further explore the lack of adaptive immunity in *Gas6*^−/−^ mice, we analyzed early immunological events after infection. For this we infected *Gas6*^−/−^ and WT mice once with *P. gingivalis* to induce an acute inflammation, and examined the development of gingival immune responses in the subsequent days (Figure 3A). First, we examined whether the expression of GAS6 and the second TAM ligand, PROS1, in the gingiva is affected by the infection. Using immunofluorescence staining on gingival histological sections and RT-*q*PCR analysis, we found that GAS6 and PROS1 were both upregulated in the oral epithelium (Figure 3B). Next, we examined by flow cytometry the content of innate myeloid leukocytes in the gingiva of the infected mice based on the gating strategy described in Figure S1 in Supplementary Material. Three days after the infection, infiltration of CD45^+^ leukocytes, particularly neutrophils and monocytes, into the infected gingiva was seen in WT mice but not in *Gas6*^−/−^ mice (Figure 3C). Of note, with regards to monocytes, a significant reduction in this population was observed in the gingiva of infected *Gas6*^−/−^ mice in comparison to the non-infected *Gas6*^−/−^ control group. We then tested gingival MHCII^+^CD11c^+^ cells representing APCs such as macrophages and DCs, the latter are expected to migrate to the draining lymph node (LN) during the three first days after infection in order to prime T cells and initiate adaptive immunity (9). Surprisingly, unlike neutrophils and monocytes, the frequencies of APCs were not reduced in the gingiva of *Gas6*^−/−^ mice after infection, and in fact, their level markedly increased in the tissue (Figures 3C,D). We thus conclude that the absence of GAS6 prevents the infiltration of myeloid leukocytes into the gingiva. In addition, the increase in the frequency of APCs together with the reduction in the monocyte population, suggests that monocytes might differentiate into APCs in *Gas6*^−/−^ mice upon infection.

### The Absence of GAS6 Facilitates Secretion of Pro-Inflammatory Molecules in the Oral Mucosa

**Figure 4:**
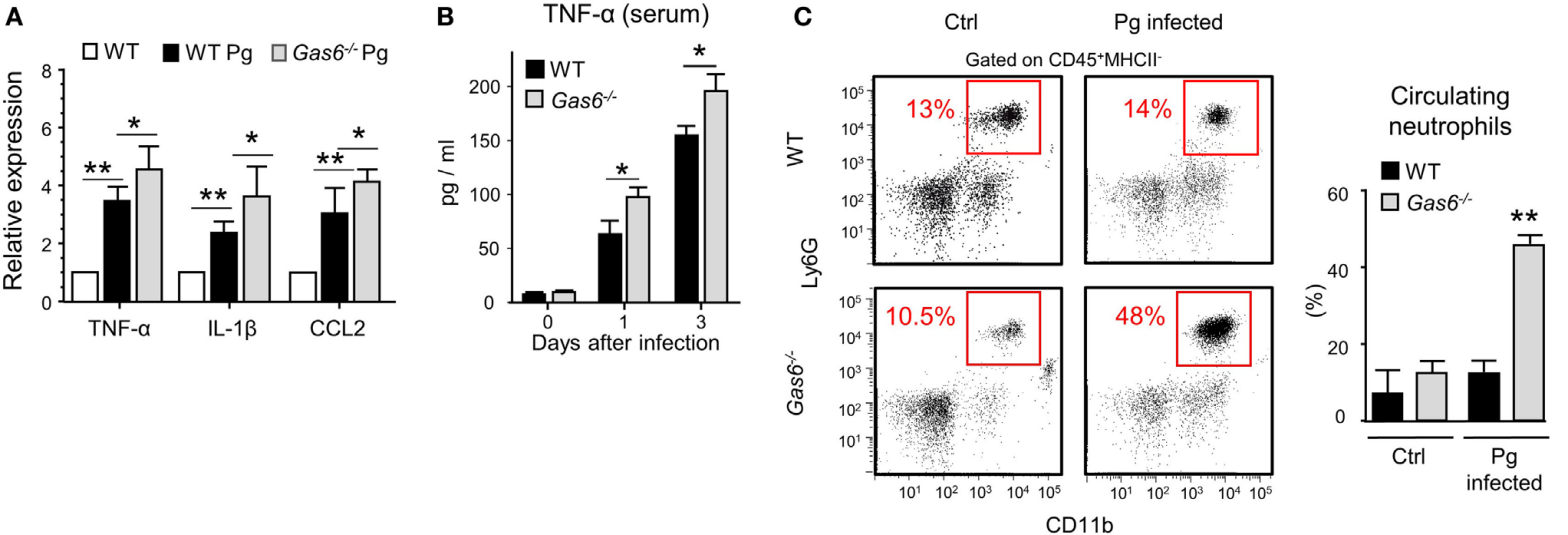
Elevated expression of pro-inflammatory cytokines in *Gas6*^−/−^ mice following infection with Pg. **(A)** Expression levels of TNF-α, IL-1β, and CCL2 in the gingiva 24 h after a single infection with Pg. Data are presented as relative mRNA expression analyzed by RT-*q*PCR and represent the mean of five mice ± SEM. **(B)** Serum levels of TNF-α, on days 0, 1, and 3 days post-infection, measured by ELISA. Graphs represent the mean of five mice ± SEM. Representative data of one out of three independent experiments is shown. **(C)** Peripheral blood samples were collected from naive and infected mice 24 h after infection for flow cytometry analysis. Representative FCAS plots and bar graphs demonstrate the percentages of neutrophils in the blood. Bar graphs represent the mean of five mice per group ± SEM. Representative data of one out of three independent experiments is shown.

The absence of inflammatory cells in the gingiva of infected *Gas6*^−/−^ mice might be due to a diminished production of inflammatory cytokines and chemokines, serving as chemoattractants for these cells. To examine this issue, we measured the gingival expression of the pro-inflammatory cytokines TNF-α and IL-1β as well as the chemokine CCL2. As depicted in Figure 4A, upon infection, both WT and *Gas6*^−/−^ mice upregulated the expression of the noted molecules; nevertheless, in *Gas6*^−/−^ mice the expression levels were significantly higher in comparison to WT mice. TNF-α in the serum of *Gas6*^−/−^ infected mice were significantly higher than WT, indicating a systemic reaction to the infection (Figure 4B). Furthermore, the percentages of neutrophils in the blood of *Gas6*^−/−^ mice were higher than those detected in infected WT mice or uninfected *Gas6*^−/−^ mice (Figure 4C). Collectively, these suggest that GAS6 in the mucosal epithelium downregulates the induction of pro-inflammatory cytokines following infection, implying that its absence should actually facilitate leukocyte infiltration upon infection with *P. gingivalis*.

### The Absence of GAS6 Prevents Leukocyte Extravasation Into the Gingiva

**Figure 5:**
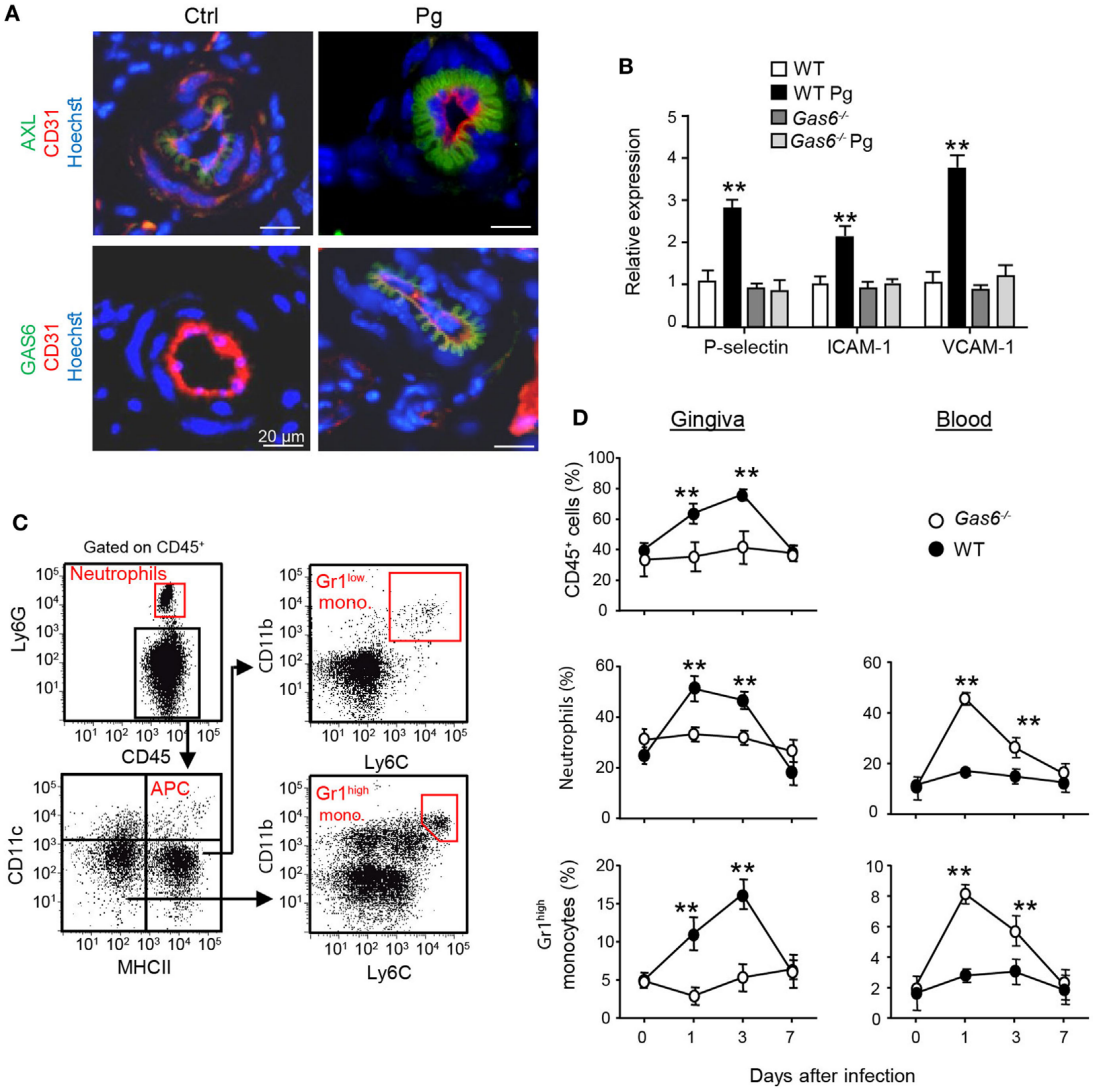
Infiltration of innate leukocytes to the gingiva is blocked in infected *Gas6*^−/−^ mice. **(A)** Immunofluorescence staining of AXL or growth arrest-specific 6 (GAS6) (green), CD31 (red), and hoechst (blue) in gingival lamina propria cross sections of *Pg*-infected and naive WT and *Gas6*^−/−^ mice. Representative images of at least three independent experiments. Scale bars represent 20 µm. **(B)** Expression of P-selectin, ICAM-1, and VCAM-1 in the gingiva of WT and *Gas6*^−/−^ mice 24 h following infection with Pg. Relative mRNA expression levels are presented using RT-*q*PCR analysis representing the mean of five mice per group ± SEM. Representative data of one out of two independent experiments is shown. **(C,D)** Quantification of inflammatory cells in the blood and gingiva following a single infection of WT and *Gas6*^−/−^ mice with Pg. **(C)** Gating strategy to identify neutrophils, APCs, Ly6C^low^, and Ly6C^high^ monocytes in the blood using flow cytometry analysis. **(D)** Time course analysis of the frequencies of CD45^+^ cells, neutrophils, and Ly6C^high^ monocytes in the blood versus gingiva in WT and *Gas6*^−/−^ mice, representing the mean of five mice per group ± SEM. Representative data of one out of two independent experiments is shown.

Another explanation to the lack of a local innate immune responses in infected *Gas6*^−/−^ mice might be the inability of inflammatory leukocytes to enter the mucosa *via* blood vessels. In this regard, it has been shown that GAS6 is required for leukocytes infiltration in systemic inflammatory models (10). Our previous work also found that GAS6 and its receptor AXL are expressed in arteries of the gingival lamina propria, while repetitive infections with *P. gingivalis* down-modulate their expression and prevents innate inflammation (6). We thus examined whether in *Gas6*^−/−^ mice the arrival of innate leukocytes to the oral mucosa is prevented after an acute infection by *P. gingivalis*. First, we analyzed by immunofluorescence analysis the expression of GAS6 and AXL in blood vessels, which were visualized using the CD31-specific antibody (Figure 5A). Whereas AXL was found in blood vessels of both naive and infected WT mice, GAS6 was only detected in infected ones. Moreover, the distribution pattern of GAS6 strikingly resembled that of AXL, suggesting that GAS6 might be bound to AXL. To further explore how GAS6 impacts the blood vessels in the oral mucosa under acute inflammatory conditions, we quantified in the gingiva the expression of endothelial adhesion molecules that are known to be upregulated upon infection. As depicted in Figure 5B, while expression of P-selectin, ICAM-1, and VCAM-1 were increased in infected WT mice, the expression of these molecules was unchanged by the infection in *Gas6*^−/−^ mice. These findings indicate that GAS6 controls leukocyte infiltration to the gingiva *via* regulating the expression of endothelial adhesion molecules. Nevertheless, since we showed above that the absence of GAS6 resulted in increased production of pro-inflammatory molecules, we analyzed changes in circulating leukocytes at various times after infection. As shown in Figures 5C,D, neutrophils and Gr-1^high^ monocytes but not DCs nor Gr-1^low^ monocytes (data not shown) accumulated in the blood of *Gas6*^−/−^ mice 1 and 3 days after infection but returned to normal levels 7 days post-infection. In WT mice, on the other hand, these cells accumulated only in the gingiva during the first 3 days post-infection, returning to basal levels by day 7. Taken together, it can be concluded that GAS6 has opposing roles during bacterial infection. Within the epithelium, upregulation of GAS6 down-modulates the secretion of pro-inflammatory cytokines and chemokines, which are required to induce egression of neutrophils and monocytes from the bone marrow (BM). However, at the same time GAS6 regulates the expression of adhesion molecules in endothelial cells and subsequently prevents leukocyte extravasation into the gingiva.

### Migration of Oral DCs to the LNs Is Accelerated by GAS6

**Figure 6:**
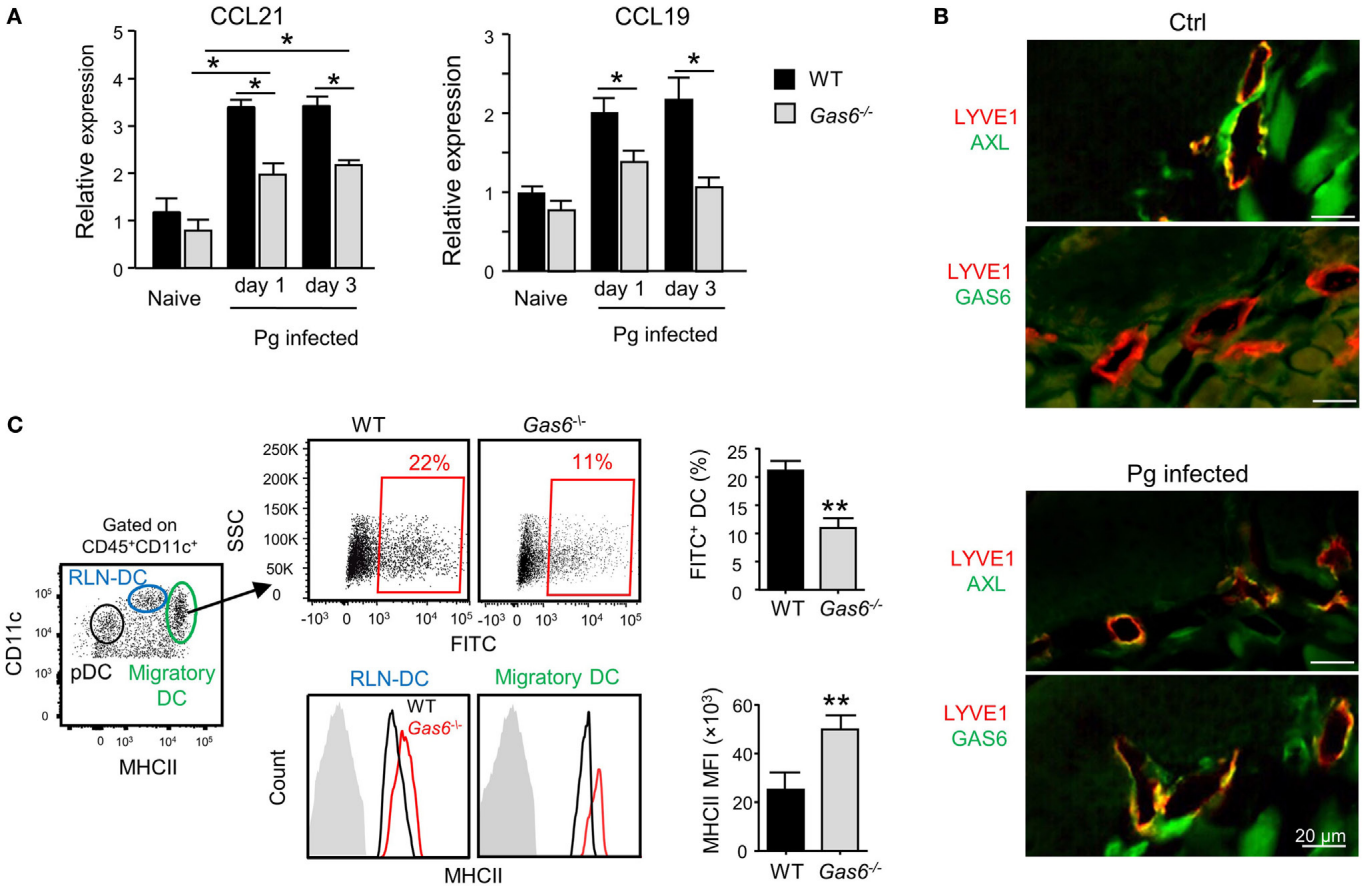
Growth arrest-specific 6 (GAS6) enhances migration of oral dendritic cells (DCs) to the draining lymph nodes (LNs). **(A)** Mice were infected once with Pg and the expression levels of CCL19 and CCL21 chemokines was analyzed in the gingiva using RT-*q*PCR 1 or 3 days later. Data represent the mean of five mice per group ± SEM. **(B)** Immunofluorescence staining of AXL or GAS6 (green) and LYVE1 (red), in gingival lamina propria cross sections of *Pg*-infected and naive WT and *Gas6*^−/−^ mice. Representative images of two independent experiments. **(C)** The oral mucosa of WT and *Gas6*^−/−^ mice were painted with FITC/DBP solution, 2 days later the draining cervical LNs were collected and processed for analysis by flow cytometry. *Left plot*—representative image demonstrating the segregation of CD45^+^CD11c^+^ cells into distinct DC subsets based on the expression of MHC class II and CD11c (RLN-DC—LN resident DCs; pDC—plasmacytoid DCs). *Upper panel*—representative FACS plots and bar graphs illustrating the percentages of FITC-positive cells among migratory DCs of *Gas6*^−/−^ and WT mice, representing the mean of five mice per group ± SEM. *Lower panel*—representative FCAS histograms showing MHCII expression levels on total migratory DCs and RLN-DCs. Gray histograms represent MHC staining on T cells which do not express MHCII. Bar graphs present the mean florescence intensity (MFI) of MHCII expression on the noted DC populations and represent the mean of five mice per group ± SEM.

The aforementioned data demonstrated that following infection higher frequencies of APCs were found in the gingiva of *Gas6*^−/−^ mice (Figure 3D). To examine whether migration of DCs to the LNs was impaired in these mice, we quantified the expression of CCL19 and CCL21, chemokines mediating DC migration to the LNs (11). Using RT-*q*PCR, we found a significant increase in the gingival expression of CCL19 and CCL21 of WT upon infection (Figure 6A). By contrast, a reduced expression of both chemokines was detected in *Gas6*^−/−^ mice 1 and 3 days after infection (Figure 6A). We next examined whether AXL and GAS6 are expressed on lymphatic endothelial cells (LECs), and if the infection alters their expression pattern. As depicted in Figure 6B, at steady state conditions AXL but not GAS6 was expressed by WT LECs that were visualized using staining with anti-LYVE-1 antibody. Upon infection, GAS6 as well as AXL were detected in LECs, suggesting that GAS6 increases permeability similar to blood endothelial cells. These results suggest that DCs might not efficiently migrate to the LNs in infected *Gas6*^−/−^ mice, due to a reduced chemokine production and impaired function of local lymphatic vessels. To address this issue directly, we painted the oral mucosa with an FITC/DBP solution, an approach allowing to track migration of oral DCs into the LNs (FITC-labeled DCs) due to local inflammation induced by the DBP (12). As demonstrated in Figure 6C, significantly less FITC-labeled DCs (gated population of CD11c^intermediate^MHCII^high^ in the LNs) were detected in *Gas6*^−/−^ compared to WT mice (~50% reduction). No FITC-labeled DCs were found in the population of LN-resident DCs (RLN) or plasmacytoid DCs, confirming that only migratory DCs originating within the oral mucosa were labeled in this assay (Figure S2 in Supplementary Material). Interestingly, despite their reduced migratory levels, DCs of *Gas6*^−/−^ mice, either migratory or RLN DCs, expressed higher levels of MHCII compared to their counterparts in WT mice (Figure 6C). Collectively, these data suggest that GAS6 facilitates DC migration to the LNs following infection, contributing to a rapid development of adaptive immunity. The augmentation in MHCII levels on DCs in *Gas6*^−/−^ mice proposes a role for GAS6 also in DC maturation and activation, which led us to explore this possibility.

### GAS6 Inhibits DC Maturation and Decreases T-Cell Activation

**Figure 7:**
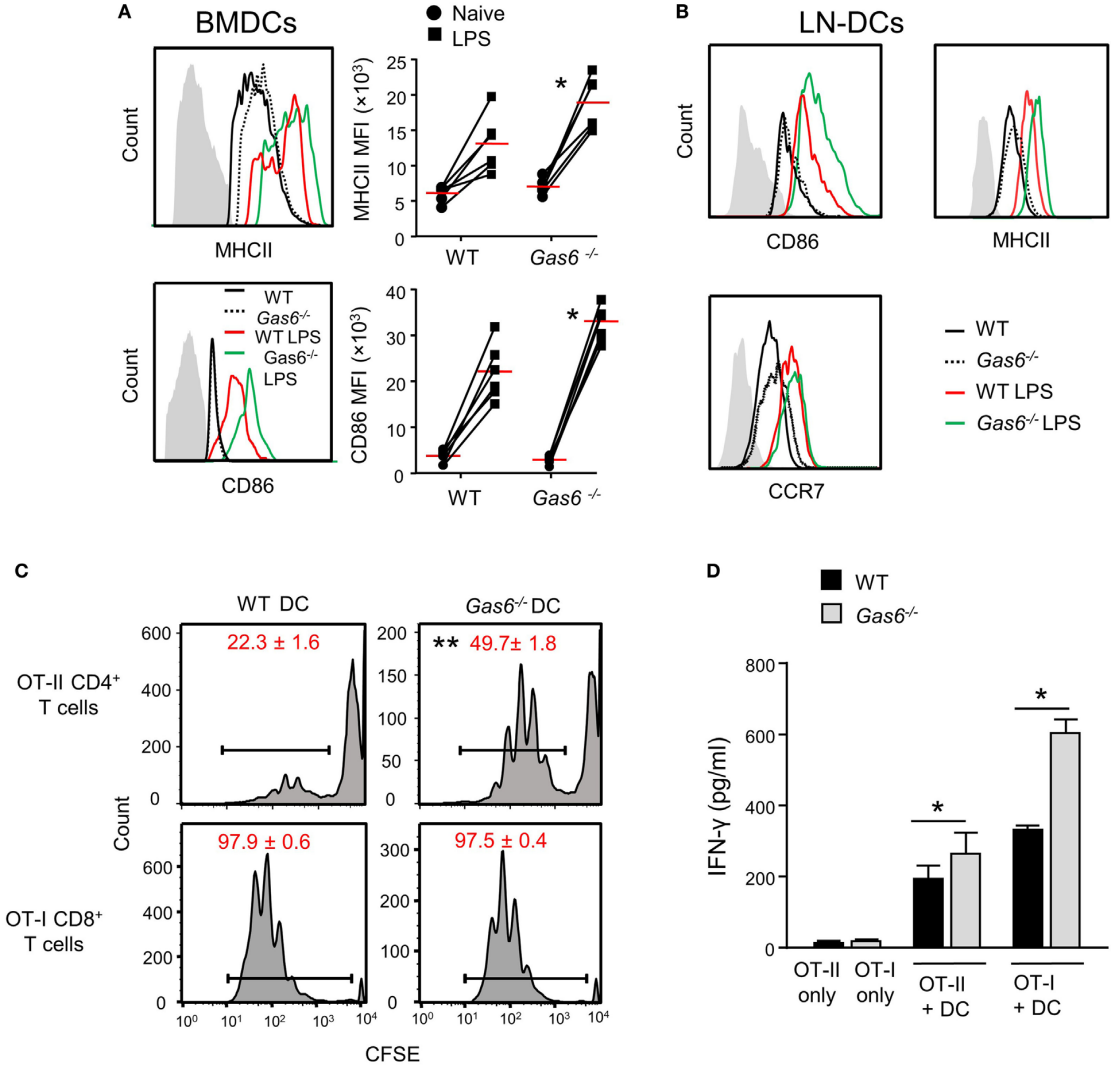
Growth arrest-specific 6 (GAS6) inhibits dendritic cell (DC) maturation and limits T-cell activation by DCs. **(A)** BMDCs generated from WT and *Gas6*^−/−^ mice were stimulated with LPS for 24 h. Representative FACS histograms and graphs demonstrating the expression and mean florescence intensity (MFI) of MHCII (top) and CD86 (bottom) on BMDCs, respectively. Gray histograms represent staining on MHC-negative cells in the culture. **(B)** DCs purified from cervical lymph nodes (LNs) of *Gas6*^−/−^ and WT mice were stimulated with LPS for 24 h. Representative FACS plots demonstrating the expression of CD86, MHCII, and CCR7 are presented. **(C,D)** DCs enriched from cervical LNs of *Gas6*^−/−^ and WT mice were stimulated with LPS and OVA peptides and then co-cultured with naive CFSE-labeled OT-II CD4^+^ T cells or OT-I CD8^+^ T cells. **(C)** Three days post-stimulation the dilution in CFSE levels on the T cells was analyzed by flow cytometry, representative FACS histograms are presented, numbers indicate the mean of three repeats per group ± SEM. **(D)** Bar graphs show the concentrations of IFN-γ secreted to the supernatant by activated CD8^+^ T cells and represent the mean of four repeats per group ± SEM. Representative data of one out of two independent experiments is shown.

We previously reported that GAS6 is expressed in DCs and its expression colocalizes with intracellularly expressed MHCII (3). We also have shown that GAS6 had no impact on surface expression levels of MHCII in DCs at steady state. These observations, together with our present finding of elevated MHCII expression after infection, suggest that GAS6 influences DC functions under inflammatory conditions. To probe this issue directly, we generated DCs from bone marrow cells (BMDCs) purified from WT and *Gas6*^−/−^ mice, and stimulated them with LPS. Using flow cytometry, we found that MHCII and CD86 expressions were upregulated upon LPS stimulation in both *Gas6*^−/−^ and WT BMDCs; yet, considerably higher levels of these molecules were found in *Gas6*^−/−^ BMDCs (Figure 7A). Similar results were obtained in DCs purified from the cervical LNs of WT and *Gas6*^−/−^ mice following LPS stimulation (Figure 7B). Interestingly, unlike MHCII and CD86, expression of CCR7 which is required for migration to the LNs was not affected by the absence of GAS6 in LN-purified DCs (Figure 7B). Next, we analyzed the ability of DCs to stimulate naive T cells. DCs were enriched from gingiva-draining cervical LNs of *Gas6*^−/−^ and WT mice, and pulsed with MHC class II-restricted OVA_223–239_ or MHC class I-restricted SIINFEKL peptides. The pulsed DCs were then co-cultured for 60 h with CFSE-labeled CD4^+^ OT-II T cells and CD8^+^ OT-I T cells, respectively. A considerable reduction in the CFSE levels was observed by all T cells indicating the capacity of the DCs to induce T-cell proliferation (Figure 7C). However, DCs lacking GAS6 had superior capacity to stimulate CD4^+^ T cells as compared to WT mice. Such an effect was not observed for CD8^+^ T cells, which upon stimulation present with proliferation levels equal to either DC population. Furthermore, we measured IFN-γ secretion in the culture supernatants by ELISA and found elevated secretion of this cytokine by CD4^+^ T cells and CD8^+^ T cells in *Gas6*^−/−^ DC cultures in comparison to the WT controls (Figure 7D). Taken together, these results demonstrate contrasting roles for GAS6 on DC functions. On one hand, GAS6 expressed by DCs downregulates their maturation and their capability to stimulate T cells. On the other hand, GAS6 enhances the expression of CCL19 and CCL21 in the tissue while not reducing CCR7 expression on activated DCs, thus facilitating DC migration to the LNs and induction of adaptive immunity. This suggests that GAS6 accelerates the induction of adaptive immunity by but also restrains its magnitude.

## Discussion

**Figure 8:**
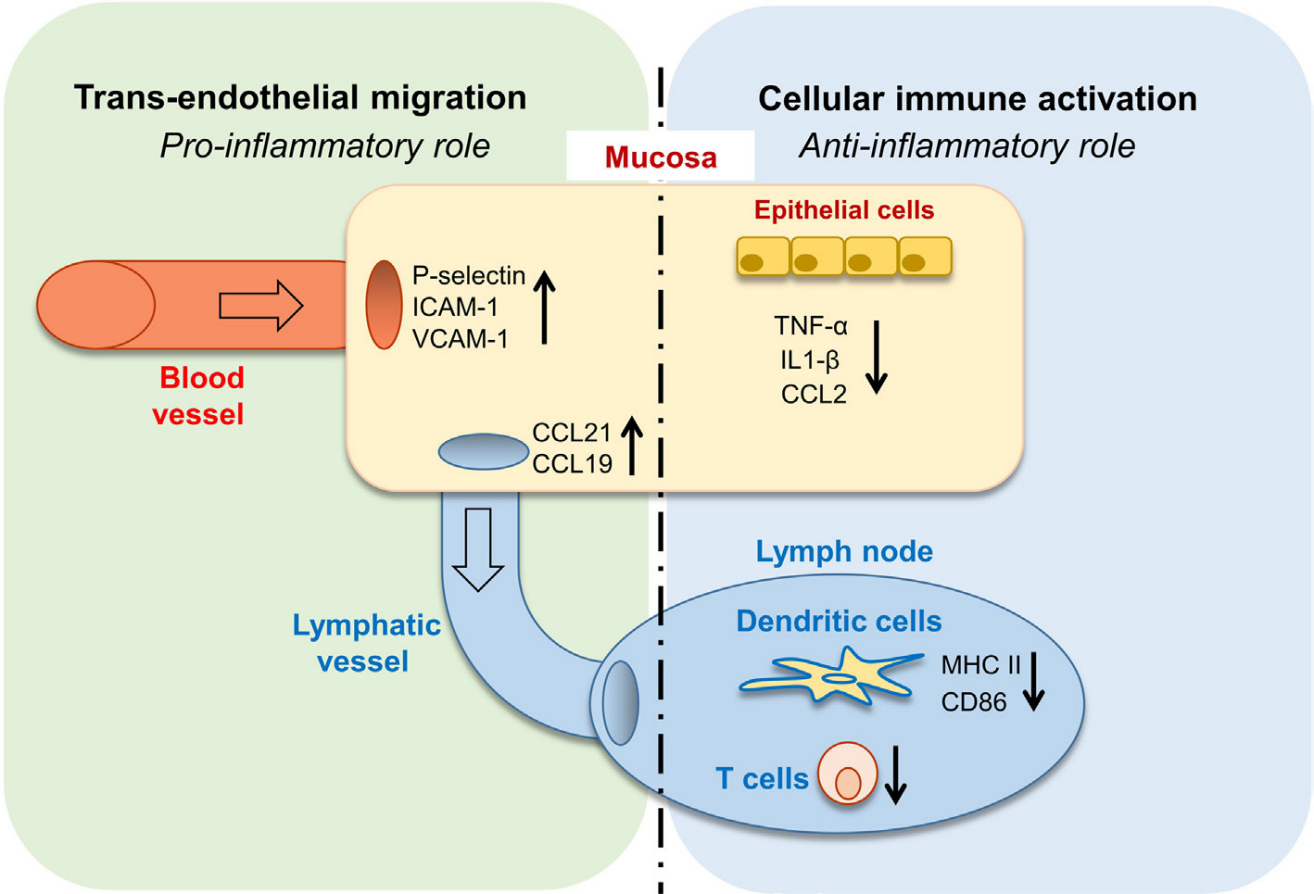
Pro- and anti-inflammatory roles for growth arrest-specific 6 (GAS6) in the oral mucosa. Upon infection, GAS6 facilitates extravasation of neutrophils and Gr1^high^ monocytes to the oral mucosa by upregulating expression of adhesion molecules on blood endothelial cells. GAS6 also enhances migration of mucosal dendritic cells (DCs) to the lymph node by upregulating CCL19/CCL21 expression, chemokines mediating this process *via* interaction with CCR7 on DCs. Both processes involve the GAS6-mediated trans-endothelial migration, indicating its pro-inflammatory function. On the other hand, GAS6 downregulates the production of pro-inflammatory cytokines and chemokines by epithelial cells. In the LN, GAS6 inhibits antigen presentation and T cell activation by DCs, *via* reducing expression of the maturation-associated molecules MHC and CD86. Interestingly, CCR7, an additional molecule associated with DC maturation, is not downregulated by GAS6, thus complementing the upregulation of CCR7 ligands by GAS6 in the mucosa to promote migration. Such apparently opposing roles of GAS6 during infection enable the induction of swift innate inflammatory responses, while facilitating the development of a restrained adaptive immune response.

In this study, we revealed the complex role of GAS6 in the oral mucosa under inflammatory conditions. Such multifaceted and apparently contrasting activities are likely attributed to the vast expression of GAS6 in various cell types within the oral mucosa. Nonetheless, it can be generalized that GAS6 positively regulates bi-directional trans-endothelial migration of leukocytes into (neutrophils and monocytes) and away (DCs) from the infected mucosa. Conversely, GAS6 negatively regulates the levels of innate and adaptive immune cells recruited to the infection site (Figure 8). Thus, integrating the overall roles of GAS6 upon infection lead us to conclude that GAS6 acts to mount a swift innate immune response and rapid initiation of adaptive immunity, while simultaneously restraining both types of responses.

Oral epithelial cells were shown to express various cytokines and chemokines such as TNF-α, IL-1β, and CCL2 that were upregulated upon infection in our study (13, 14). GAS6 is constitutively expressed in oral epithelial cells and as we recently showed it downregulates the activation of epithelial cells at steady state in order to maintain homeostasis (3). Here, we demonstrate that expression of GAS6 is increased rapidly under inflammatory conditions to inhibit excessive activation of the epithelium, which as we previously reported might have pathological consequences (6). Similar to epithelial cells, GAS6 is also constitutively expressed by macrophages and regulates their steady state function (15). However, upon TLR-mediated activation, expression of GAS6 is decreased enabling the macrophages to mount a potent secretion of pro-inflammatory cytokines and chemokines. These findings further emphasize the versatile functions of GAS6 in immunoregulation and highlight that GAS6 acts in a cell-specific fashion.

Our analysis reveals that GAS6 regulates CCL21 and CCL19 expression in the gingiva and in gingival lymphatic vessels under inflammatory conditions. In contrast to its inhibitory effect on MHCII and CD86 expression on stimulated DCs, GAS6 does not downregulate the expression of their receptor CCR7 on these cells. This demonstrates that the versatile functions of GAS6 in epithelial cells, LECs, and DCs are critically orchestrated to facilitate migration of mature DCs to the LNs *via* the afferent lymphatic vessels (16). To the best of our knowledge, this is the first observation linking GAS6 with activation of the lymphatic system. This lymphatic expression pattern of GAS6 resembles its expression in blood endothelial cells, leading to expression of adhesion molecules. Since LEC also express VCAM-1 and ICAM-1 (17), which were upregulated in the gingiva of infected *Gas6*^−/−^ mice, it is likely that GAS6 also enhances DC migration by enabling endothelial attachment of mature DCs. With regards to chemokine expression, CCL21 is known to be expressed by LECs, and therefore it is likely that GAS6 activates LECs similar to its role on blood endothelial cells. CCL19, on the other hand, is not expressed by LEC but rather by activated DCs, and was proposed to induce their homing to the lymphatics (18). The possibility that in our study GAS6 upregulates CCL19 expression in oral DCs is in line with its incapability to reduce their CCR7 expression (in contrast to the other maturation markers MHCII and CD86), as both CCL19 and CCR7 are required for DC migration to the LNs. In a broader view, the impact of GAS6 on activated DCs suggests that migratory capability (represented by CCL19 and CCR7) and antigen-presentation function (represented by MHCII and CD86) of mature DCs are differentially regulated. In a recent study, PROS1 expressed by T cells was shown to reduce antigen presentation by DCs and to subsequently limit adaptive immunity (19). Here, we showed that DC maturation and antigen presentation is regulated by GAS6 expressed in DCs, thus TAM ligands appear to tightly control adaptive immunity by acting *via* both T cells and DCs.

*In vitro* stimulation of naive CD4^+^ T cells but not CD8^+^ T cells by *Gas6*^−/−^ DCs resulted in a decreased proliferative capacity in comparison to WT DCs. This could be explained by the considerable downregulation of MHCII but not MHCI in *Gas6*^−/−^ DCs upon activation. Nevertheless, in both T cell types, the capability of the cells to secrete IFN-γ was significantly reduced due to the absence of GAS6 in DCs. A recent study has shown that AXL signaling downregulates IL-12 production by DCs (20), a cytokine required for generation of IFN-γ producing T cells (21). In addition, the AXL/GAS6 pathway was shown to diminish IFN-γ expression in NK cells as well as the expression of its master regulator, T-BET (22). T-BET is a transcription factor that is crucial for the induction of Th1-type immune responses and also for cytotoxic CD8^+^ T cells (23). It is thus possible that GAS6 can regulate T-cell polarization during their priming by DCs.

In summary, this study demonstrates that GAS6 exerts both pro- and anti-inflammatory responses in the oral mucosa to ensure rapid but restrained pathogen-specific immunity. Together with the previously reported capability of GAS6 to control oral homeostasis (3), it can be concluded that GAS6 is a key regulator of the oral mucosal immune system.

## Materials and Methods

### Mice

*Gas6*^−/−^ (24) and *Gas6^+/+^* littermate controls (WT) were prepared by crossing *Gas6*^−/−^ mice and C57BL/6 (B6) purchased from Harlan (Rehovot, Israel). The identity of the mice used for experiments was confirmed by genotyping with the following PCR primers: forward-GAGTGCCGTGATTCTGGTC, middle-CCACTAAGGAAACAATAACTG, and reverse-ATCTCTCGTGGGATCATT. The mice were maintained under SPF conditions and analyzed between 8 and 12 weeks of age.

### Antibodies and Reagents

The following fluorochrome-conjugated monoclonal antibodies and the corresponding isotype controls were purchased from BioLegend (San Diego, CA, USA): I-A/I-E (M5/114.15.2), CD45.2 (104), CD86 (PO3), Ly6G (1A8), Ly6C (HK1.4), CD3 (17A2), CD11b (M1/70), CD8α (53-6.7), CD4 (GK1.5), FOXP3 (MF-14), and CD11c (N418). CFSE was purchased from Molecular Probes. *E. coli* LPS were purchased from Sigma (Israel).

### Isolation and Processing of Gingival Tissue

Gingival tissues were excised, minced, and treated with Collagenase type II (2 mg/ml; Worthington Biochemicals) and DNase I (1 mg/ml; Roche) solution in PBS plus 2% fetal calf serum (FCS) for 25 min at 37°C in a shaker bath. A total of 20 µl of 0.5 M EDTA per 2 ml sample was added to the digested tissues and incubated for an additional 10 min. The cells were washed, filtered with a 70 µm filter, and stained with antibodies as indicated in the text. In some experiments, FOXP3 staining was performed using the FOXP3 Fix/Perm Buffer Set (BioLegend) according to the manufacturer’s instructions. The stained samples were run in the LSR II (BD Biosciences) flow cytometer and analyzed using FlowJo software (Tree Star).

### Immunofluorescence Staining

The maxilla was fixed overnight at 4°C in 4% paraformaldehyde/PBS solution, and then washed for 1 week in EDTA 0.5 M/PBS that was changed every other day. The tissue was cryopreserved in 30% sucrose (overnight at 4°C), embedded in OCT, and cryosectioned into 10 µm-thick sections. The cross sections, as well as the separated epithelium, were washed three times in PBS, blocked in a blocking buffer (5% FCS, 0.1% Triton X-100 in PBS) for 1 h at room temperature, and incubated with primary antibodies: goat anti-GAS6 (clone sc-1935, Santa Cruz Biotechnology), rabbit anti-PROS1 (clone 2428718, Millipore), rat anti-CD31 (clone 550274, BioLegend), goat anti-AXL (clone sc-1096, Santa Cruz Biotechnology), and rabbit-Anti-LYVE1 (clone-ab14917, Abcam) overnight at 4°C. Following three washing steps in PBS, the samples were incubated with a secondary antibody: donkey anti-goat IgG, donkey anti-rat IgG, or donkey anti-rabbit IgG (Jackson ImmunoResearch) diluted 1:100 in blocking buffer for 1 h at RT, washed three times, stained with hoechst, and mounted. As a negative staining control, primary antibody was omitted and replaced by blocking buffer. Signals were visualized and digital images were obtained using an Olympus BX51 fluorescent microscope mounted with a DP72 (Olympus) camera.

### RNA Extraction and RT-*q*PCR

For RNA isolation, the maxilla was homogenized in 1 ml TRI reagent (Sigma) using electric homogenizer, and RNA was extracted according to the manufacturer’s instructions. cDNA synthesis was performed using the qScript™ cDNA Synthesis Kit (Quanta-BioSciences Inc™). Real-Time *q*PCR reactions (20 µl volume) were performed using Power SYBR Green PCR Master Mix (Quanta-BioSciences Inc™). The following reaction conditions were used: 10 min at 95°C, 40 cycles of 15 s at 95°C, and 60 s at 60°C. The samples were normalized to the TBP (TATA box binding protein) as control mRNA, by change in cycling threshold (ΔC_T_) method and calculated based on 2^−ΔΔCT^.

### Inflammation-Induced Bone Loss Model

Mice were treated with 0.4% trimethoprim and sulfamethoxazole solution (Resprim; Teva) in the drinking water for 10 days, followed by 3 days without antibiotics. The mice were then infected *via* oral gavage, three times at 2-day intervals, with 1 × 10^10^ cfu of *P. gingivalis* 53977 in 400 µl of 2% (wt/vol) CMC solution (Sigma). Uninfected mice were treated with the CMC vehicle alone. Six weeks later, the mice were euthanized and the hemi-maxillae were harvested and scanned using μCT (Scanco Medical). 3D alveolar bone loss was quantified as we previously described (9).

### Acute Infection With *P. gingivalis*

Mice were infected *via* oral gavage once with 1 × 10^10^ cfu of *P. gingivalis* 53977 in 400 µl of 2% (wt/vol) CMC solution. At days 1, 3, and 7 post-infection, the gingiva and blood samples were collected from the mice for analysis.

### Serum Analysis

Six weeks after infection, blood was drawn from the mice and the sera were stored at −80°C. Ninety-six-well plates (Nunc) were coated overnight at 4°C with 1 µg of *P. gingivalis* 53977 lysate/well in 0.1 M bicarbonate buffer (pH 9). The plates were washed twice with PBS-0.02% Tween 20 and blocked with PBS 10% FCS (2 h at room temperature). Subsequently, mouse serum samples diluted serially in PBS were added to the wells for 3 h incubation at RT. This was followed by four washes in PBS-0.02% Tween 20 and the addition of anti-mouse peroxidases-conjugated IgG, IgG1, and IgG2c antibodies (Jackson ImmunoResearch). After incubation for 2 h at RT, the plates were washed five times and 100 µl/well of tetramethyl benzidine (TMB) solution (Southern Biotech) was added for 5 min, followed by the addition of 100 µl of TMB stop solution (Southern Biotech). Absorption was read at 450 nm using an iMARK microplate reader (Bio-Rad).

### T-Cell Activation Assay *Ex Vivo*

Cervical LNs were collected from WT or *Gas6*^−/−^ mice and treated with collagenase type II (1 mg/ml) and DNase I (1 mg/ml) solution in PBS plus 2% FCS for 20 min at 37°C in a shaker bath. 20 µl of 0.5 M EDTA per 2 ml sample was added to the digested LNs and incubated for an additional 10 min. The cells were then washed and filtered. CD11c^+^ cells were enriched from the digested LNs by positive isolation using MACS MicroBeads according to the manufacturer’s instructions (Miltenyi Biotec, Germany). OT-I CD8^+^ T cells or OT-II CD4^+^ T cells were purified by negative selection with the EasyStep mouse CD8^+^ or CD4^+^ T-cell enrichment kits, respectively, according to the manufacturer’s instructions (StemCell Technologies, Canada). The purified T cells were incubated with same volume of 5 mM CFSE in HBSS 10 min at 37°C at a final concentration of 2.5 mM. Labeling was quenched by adding an excess of ice-cold RPMI 1640 complete medium, and cells were washed twice with culture medium. CFSE-labeled OTI CD8^+^ or OT-II CD4^+^ T cells (5 × 10^4^/well) were incubated with the enriched CD11c^+^ cells (1 × 10^4^/well) in 96-well U-bottom plates (Nunc). The SIINFEKL (1 µg/ml) and OVA_223–339_ (1 µg/ml) peptides were added to cultures containing CD8^+^ or CD4^+^ T cells, respectively, in the presence of LPS (Sigma) (100 ng/ml). The cultures were then incubated for 60 h, the supernatants were collected and stored at −80°C immediately, and the dilution of CFSE fluorescence in the cell fraction was analyzed using an LSR II instrument.

### Preparation and Stimulation of BMDCs

The femur was isolated from the mice, cleaned from soft tissues in RPMI 1640, and soaked in 70% ethanol for 1 min for sterilization. The femur was then washed with sterile PBS and the bone ends was removed by sterile scissors. BM cells eluted from the bone by flushing them several times using sterile syringe filled with RPMI 1640, and the cells were then washed, treated with ACK solution for 3 min on ice, washed again, and counted. BM cells (5 × 10^5^ cells/well) in 24-well plates (Nunc) were cultured with complete RPMI media [450 ml RPMI 1640, 50 ml FCS, 5 ml l-glutamine, 50 µM β-mercaptoethanol, penicillin (100 U/ml), streptomycin (100 µg/ml), and gentamicin (50 µg/ml)] supplemented with GM-SCF (20 ng/ml) for 4 days to induce their differentiation into BMDCs (CD11C^+^MHCII^+^). The cultures were then exposed to LPS (Sigma) (100 ng/ml), washed, and stained with the noted antibodies for flow cytometry analysis.

### Splenocytes Restimulation and Quantification of IFN-γ Secretion

Splenocytes were prepared from the various groups of mice, plated in a concentration of 1 × 10^6^ cells/well and RgpA (1 µg/ml) was added for 3 days. Supernatants were then collected and the level of IFN-γ in the supernatants was measured using an ELISA MAX mouse IFN-γ kit (BioLegend) according to the manufacturer’s instructions. Cytokine levels were determined using standard curves of recombinant cytokines and are expressed as picogram per milliliter.

### FITC Painting

FITC (Sigma) was dissolved as a 20% (w/v) solution in DMSO (Sigma-Aldrich) and then diluted to 2% (v/v) FITC solution prepared in acetone and dibutyl phthalate (DBP; 1:1). Mice were painted on both sides of the buccal mucosa 2 days before harvesting the draining LNs.

### Statistical Analysis

Data were expressed as mean ± SEM. Statistical tests were performed using one-way analysis of variance and Student’s *t* test. *P* < 0.05 was considered significant. *P < 0.05, **P < 0.005.

## Ethics Statement

This study was carried out in accordance with the recommendations of the IACUC of the Hebrew University. The protocol was approved by the Hebrew University Institutional Animal Care and Ethics Committee.

## Author Contributions

MN, AW, TB-C, and A-HH designed the research; MN, GM, YT, TC, and LE-B analyzed the data; MN, GM, TN, FS, RS, and LE-B performed the experiments; A-HH and TB-C wrote the manuscript; AHH and TB-C: funding acquisition.

## Conflict of Interest Statement

The submitted work was carried out without the presence of any personal, professional, or financial relationships that could potentially be construed as a conflict of interest.
