# Supplementary figures and images for: Multiple Regulatory Levels of Growth Arrest-Specific 6 in Mucosal Immunity Against an Oral Pathogen
Source: Front Immunol. 2018 Jun 18;9:1374. doi: 10.3389/fimmu.2018.01374 (PMC6015888; doi:10.3389/fimmu.2018.01374)

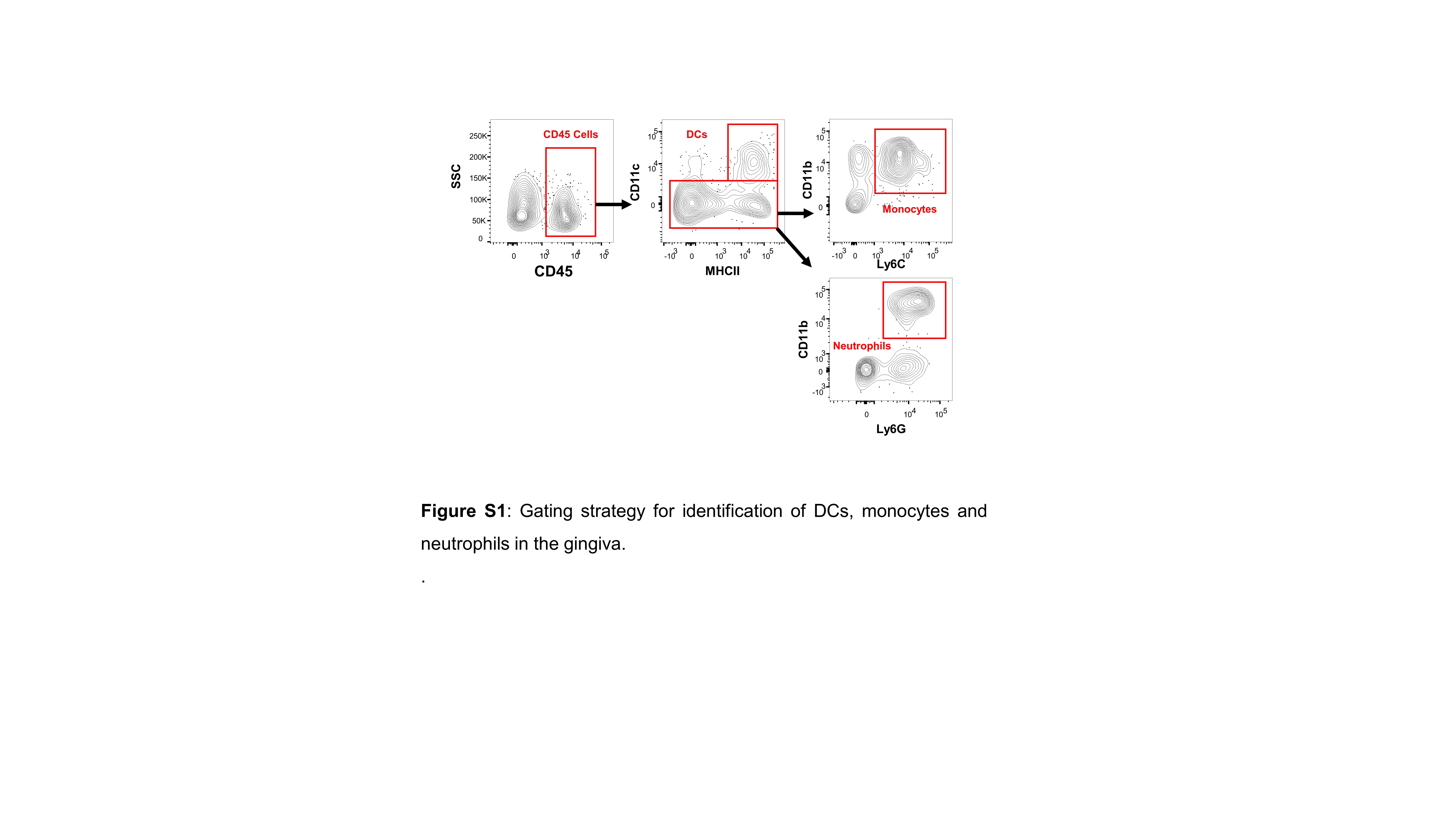

Supplement: Supplementary file 1 [file image_1.jpeg]

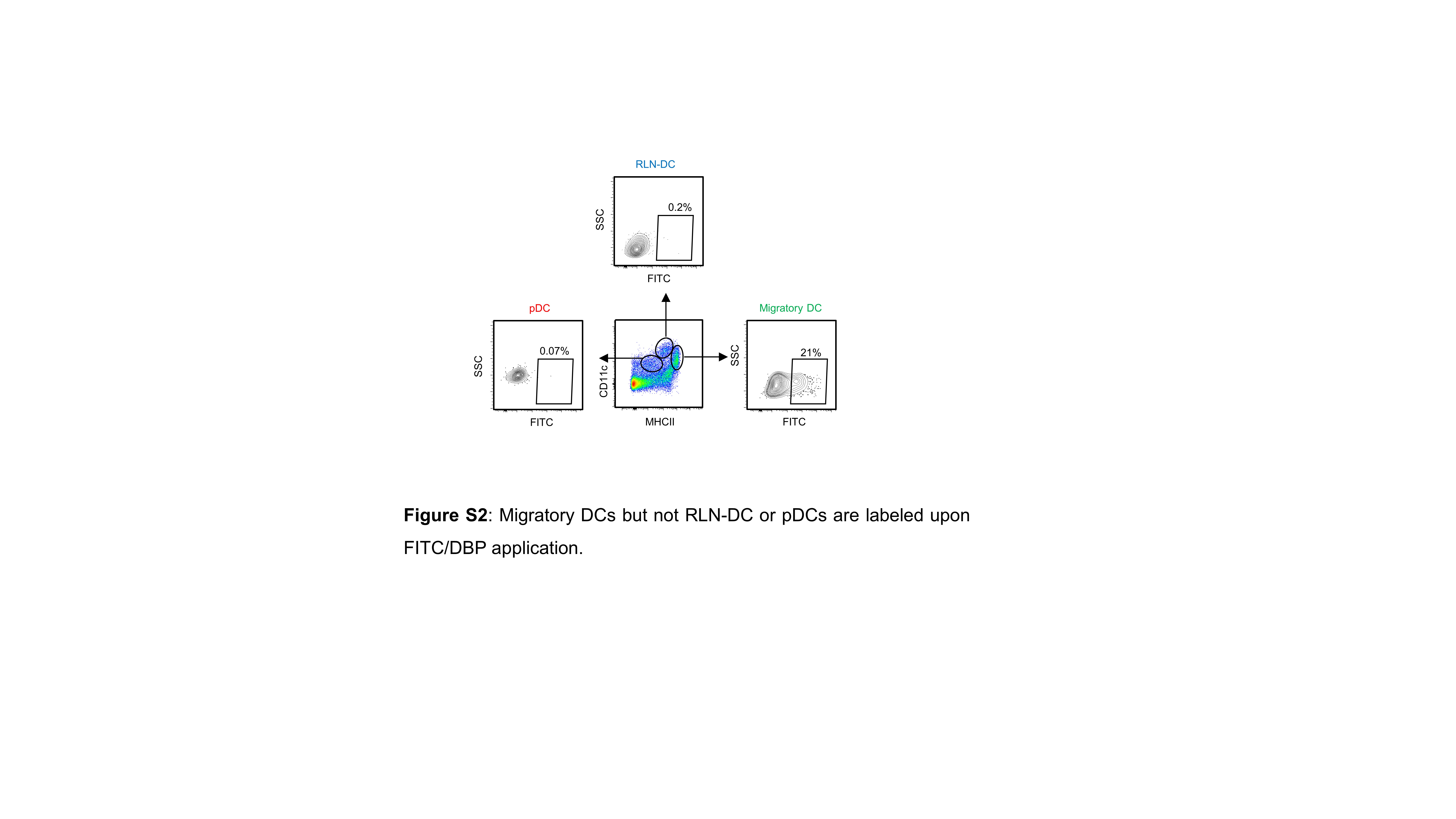

Supplement: Supplementary file 2 [file image_2.jpeg]
